# Tight coupling of polymerization and depolymerization of polyhydroxyalkanoates ensures efficient management of carbon resources in *Pseudomonas putida*

**DOI:** 10.1111/1751-7915.12040

**Published:** 2013-02-28

**Authors:** Sagrario Arias, Monica Bassas-Galia, Gabriella Molinari, Kenneth N Timmis

**Affiliations:** 1 Environmental Microbiology Laboratory, Helmholtz Centre for Infection ResearchInhoffenstrasse 7, D-38124, Braunschweig, Germany; 2 Institute for Microbiology, Technical University BraunschweigSpielmannstrasse 7, D-38106, Braunschweig, Germany

## Abstract

Environmental microbes oscillate between feast and famine and need to carefully manage utilization, storage and conversion of reserve products to exploitable sources of carbon and energy. Polyhydroxyalkanoates (PHAs) are storage polymers that serve bacteria as sources of food materials under physiological conditions of carbon demand. In order to obtain insights into the role of PHA depolymerase (PhaZ) and its relationship to a PHA polymerase (PhaC2) in the carbon management activity of *Pseudomonas putida* strain U, we created a polymerase hyperexpression strain and a depolymerase knockout mutant of this strain, and examined their synthesis of PHA and expression of their PHA genes. This study revealed that hyperexpression of PhaC2 led to the accumulation of higher amounts of PHA (44%wt) than in the wild-type strain (24%wt) after 24 h of cultivation, which then returned to wild-type levels by 48 h, as a result of elevated depolymerization. The *phaZ* mutant, however, accumulated higher levels of PHA than the parental strain (62%wt), which were maintained for at least 96 h. Transcriptional analysis of the *pha* cluster by RT-PCR revealed that PHA operon proteins, including depolymerase, are expressed from the beginning of the growth phase. Hyperexpression of the PhaC2 polymerase was accompanied by an increase in the expression of the PhaZ depolymerase and a decrease in expression of another PHA polymerase, PhaC1. This suggests tight regulatory coupling of PHA polymerase and depolymerase activities that act in synergy, and in concert with other PHA proteins, to provide dynamic PHA granule synthesis and remodelling that rapidly and sensitively respond to changes in availability of carbon and the physiological-metabolic needs of the cell, to ensure optimal carbon resource management.

## Introduction

All free-living organisms practice carbon resource management to an extent that is possible. Whereas many animals and plants generally regulate carbon uptake to match metabolic needs, other organisms, particularly opportunistic environmental microbes subjected to widely fluctuating carbon availability, may assimilate all carbon that is capturable and manage its utilization through consumption and growth, on one hand, and conservation by conversion to storage polymers, on the other (Reis *et al*., 2003). Interconversions between readily metabolizable and more inert intracellular storage products are central to this. Even organisms that regulate carbon uptake exploit such interconversions for fine tuning of their carbon management, in order to optimize their cellular metabolic networks and their population-level ecophysiological processes.

Widely exploited storage products in the microbial world are polyhydroxyalkanoates (PHAs), wax esters and triacylglycerols, which are formed into characteristic intracellular hydrophobic granules (Haywood *et al*., 1990; Huisman *et al*., 1991; Timm and Steinbüchel, 1992; Kim *et al*., 2007; Arias and Bassas, 2010). Such storage products are typically produced under conditions of carbon availability when growth is otherwise restricted because of limitation of another essential nutrient, such as nitrogen or oxygen (Madison and Huisman, 1999; Luengo *et al*., 2003; Velázquez *et al*., 2007). Carbon is then channelled less towards metabolism and growth, and more towards storage products. Food reserves are subsequently mobilized for cellular maintenance under environmental conditions allowing no or slow growth, or for growth when conditions of nutrient balance return. It is to be expected that the competitive ability of microbes in changing environments is to some extent determined by how nimble they are in storing excess carbon, and especially in mobilizing their storage products, as a function of the metabolic status of the cell and the prevailing fluxes of nutrients needed for growth and development (Hoffmann and Rehm, 2005; Ciesielski *et al*., 2010). The opposing processes of storage product polymerization and depolymerization, their regulation, and the integration of their regulation in the metabolic and regulatory network hierarchy of the cell, are thus pivotal to optimal cellular function and organism-population competitivity.

A key issue in this context is: how does the cell assure optimal activities of opposing reactions while avoiding futile cycles? Part of the answer may lie in the spatial sequestration/compartmentalization of competing activities within the polymer granule, which is not simply a solid amorphous mass of polymer but rather a structured heterogeneous body containing polymerase(s) and depolymerase(s), other PHA-relevant enzymes, and structure-orchestrating proteins, like phasins (Fig. S1A) (Prieto *et al*., 1999a; York *et al*., 1995; Pötter and Steinbüchel, 2005; Neumann *et al*., 2008). But part of the answer also lies in the relative amounts of polymerase and depolymerase in the granule, which are determined to a large extent by their regulated production (Uchino *et al*., 2007; Ren *et al*., 2009a; de Eugenio *et al*., 2010a,2010b). Thus far, the factors controlling the processes of polymerization and depolymerization are still poorly understood; their characterization will not only lead to a better understanding of carbon management by the cell, and hence the biology of the system, but should also provide a basis for the development of improved systems for biotechnological production of storage polymers as alternatives to petrochemical-based polymers.

In the present study, we investigated regulatory interactions of the PhaC2 synthase-polymerase and the PhaZ depolymerase of *Pseudomonas putida* strain U (PpU), through the generation of hyperproduction and deletion mutants, employing a chromosomally integrated expression vector, and analysis of their transcription phenotypes and PHA accumulation properties.

## Results

### Hyperexpression of the PhaC2 PHA synthase results in increased PHA accumulation

We designed a bipartite, mini-transposon-based hyperexpression system for the PpU PhaC2 synthase, consisting of (i) a specialized mini-Tn*5*, pCNB1*xylS*/*Pm*::*T7pol*, expressing T7 polymerase from the XylS3-metylbenzoate (3-MB)-regulated promoter *Pm*; and (ii) a hybrid pUT-miniTn*5*-Tel-derivative expressing the *phaC2* gene from the T7 polymerase promoter (Fig. S1B). The two mini-transposon components were separately and randomly inserted into the PpU chromosome. The best PHA producer was selected after two rounds of screening, involving semi-quantification of PhaC2 production by SDS-PAGE separation of cellular proteins and inspection of PHA granule formation by fluorescence microscopy of Nile Red-stained cells (data not shown). This strain was designated PpU 10–33.

**Figure 1:**
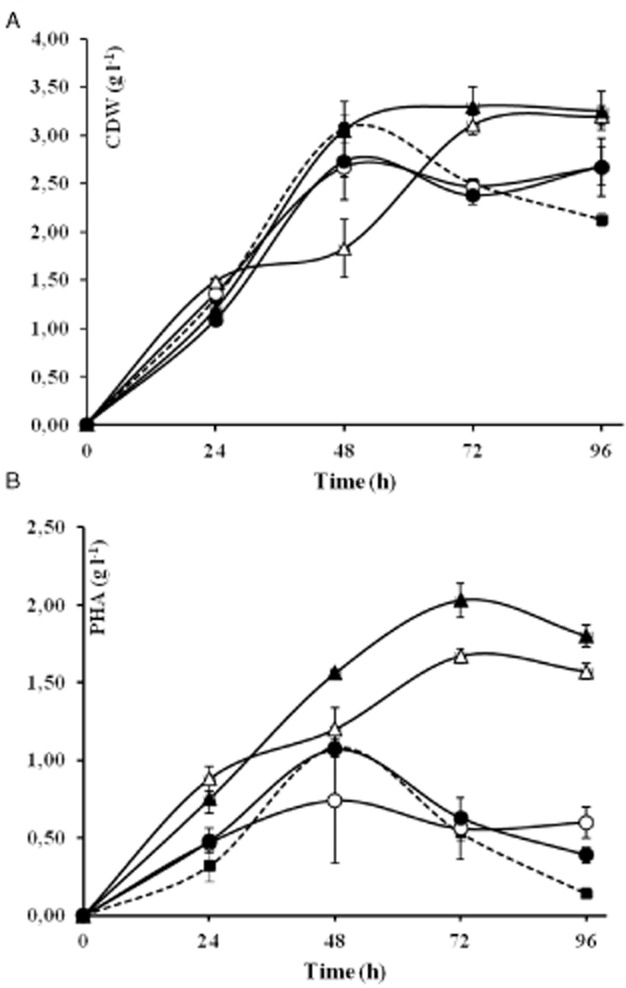
PHA production in *P. putida* U and the recombinant strains. Strains were cultured in modified MM with sodium octanoate 35 mM and were induced (I) with 0.5 mM 3-MB or not induced (NI). (A) shows the biomass yields and (B) PHA accumulation (g l^−1^) for PpU (filled squares), PpU 10–33 NI (open circles), PpU 10–33 I (filled circles), and PpU 10–33-Δ*phaZ* NI (open triangles) and I (filled triangles). Values are means of duplicates and/or triplicates and errors were calculated with the SEM function by using the GraphPad Prism statics program.

Figure 1 and Table S1 show the growth and PHA yields of PpU 10–33 and its parental strain PpU over time in cultures in modified MM medium with sodium octanoate given in two pulses of 15 and 20 mM, the second pulse given at the time of induction. As can be seen, peak biomass and PHA levels were reached at 48 h (Fig. 1B, Table S1B). PHA levels in the hyperexpressing strain were around 50% higher than those in the parental strain at 24 h but were around 25% lower than those of the parental strain at 48 h and similar at 72 h, suggesting that an increase in PhaC2 causes a transient increase in PHA, which in turn provokes an increase in depolymerization activity until levels are normalized. Importantly, the PHA percentage of cellular dry weight (%wt) dropped precipitously after 48 h from 35% to 7%wt, in the case of PpU, and from 39% to 15%wt, in the case of PpU 10–33 induced cultures.

The reason why non-induced cultures of PpU 10–33 also showed a 50% increase in PHA accumulation over that of the wild-type strain at 24 h was not investigated further, but was assumed to reflect leakiness of the T7 promoter (also indicated by RT-PCR results; see below).

The highest biomass levels, 3.07 g l^−1^ in the case of PpU, and 2.67 g l^−1^ (uninduced, NI) and 2.73 g l^−1^ (induced, I) in the case of PpU 10–33 (Fig. 1A, Table S1A), and PHA accumulation, 1.08 g l^−1^, 0.74 g l^−1^ and 1.07 g l^−1^, respectively (Fig. 1B, Table S1B), were attained at 48 h of cultivation with both strains. After 48 h, biomass and PHA levels dropped, with PHA levels diminishing or falling more significantly than biomass levels. The PpU 10–33 strain gave higher yields of PHA, expressed as percentage of biomass, at almost all sampling times. The highest PHA yield measured in this experiment, 44%wt, was obtained in PpU 10–33 induced cells at 24 h, compared to 24%wt in PpU and 35%wt in uninduced PpU 10–33 cells (Table S1B). At 48 h, when the highest biomass yield was obtained, the highest absolute yield, 41% of cellular dry weight (CDW) of PHA, was obtained in uninduced cells of 10–33, compared with 35 in PpU and 40% wt in induced PpU 10–33 cultures. Thus, the effect of induction is seen primarily in relatively young cultures. Importantly, the percentage of PHA dropped precipitously after 48 h to 7%wt in the case of PpU and 15–22%wt in the case of PpU 10–33.

### Inactivation of the PhaZ depolymerase results in higher PHA peak levels that are maintained over time

A *phaZ* deletion mutant of the PpU 10–33 strain, designated PpU 10–33 Δ*phaZ*, was created (see *Experimental procedures*) and subsequently assessed for PHA accumulation. As can be seen in Fig. 1 and Table S1, cultures of the mutant exhibited higher PHA levels (62%wt) and, in contrast to the situation with the PhaZ-producing strains, these levels were maintained until at least 96 h of cultivation. Thus, the Δ*phaZ* knockout phenotype suggests that the PhaZ depolymerase is a major determinant of PHA accumulation and maintenance in the cell.

In order to causally relate the *phaZ* gene mutation to the observed phenotype, and to rule out any indirect effects on expression of the *pha* cluster, the *phaZ* gene was PCR-amplified, cloned in the pBBR1MCS-5 plasmid vector, and introduced into the PpU 10–33-*ΔphaZ* strain. PHA production and maintenance in the complemented mutant, PpU 10–33-Δ*phaZ* pMC-*phaZ*, designated strain pMC-*phaZ*, was then assessed. Table 1 shows the biomass and PHA yields of the PpU 10–33 strain, its *phaZ* deletion mutant and the complemented derivative, after growth for 44 h in modified MM with sodium octanoate (20 mM). Biomass yields for the three stains were similar at about 2 g l^−1^ whereas PHA yields were 21%wt for the PpU 10–33 strain, 41%wt for its Δ*phaZ* mutant, and 5%wt for the complemented strain. The lower than wild-type levels of PHA in the complemented strain presumably reflects higher cellular depolymerase levels, resulting from the complementing gene being located on a multicopy vector.

**Table 1:** Effect of *phaZ* gene deletion on PHA yields in PpU 10–33

Strains	CDW (g l^−1^)	PHA (g l^−1^)	PHA (% wt)
PpU 10–33	2.11	0.45	21.0
PpU 10–33- Δ*phaZ*^a^	2.18	0.90	41.0
pMC-PhaZ^b^	1.98	0.10	5.0

Strains were cultivated in modified MM with 20 mM octanoate for 44 h, without the addition of 3-MB inducer.

a Knockout strain of the *phaZ*.

b Complemented strain.

### PHA granule morphology is different in the constructs

**Figure 2:**
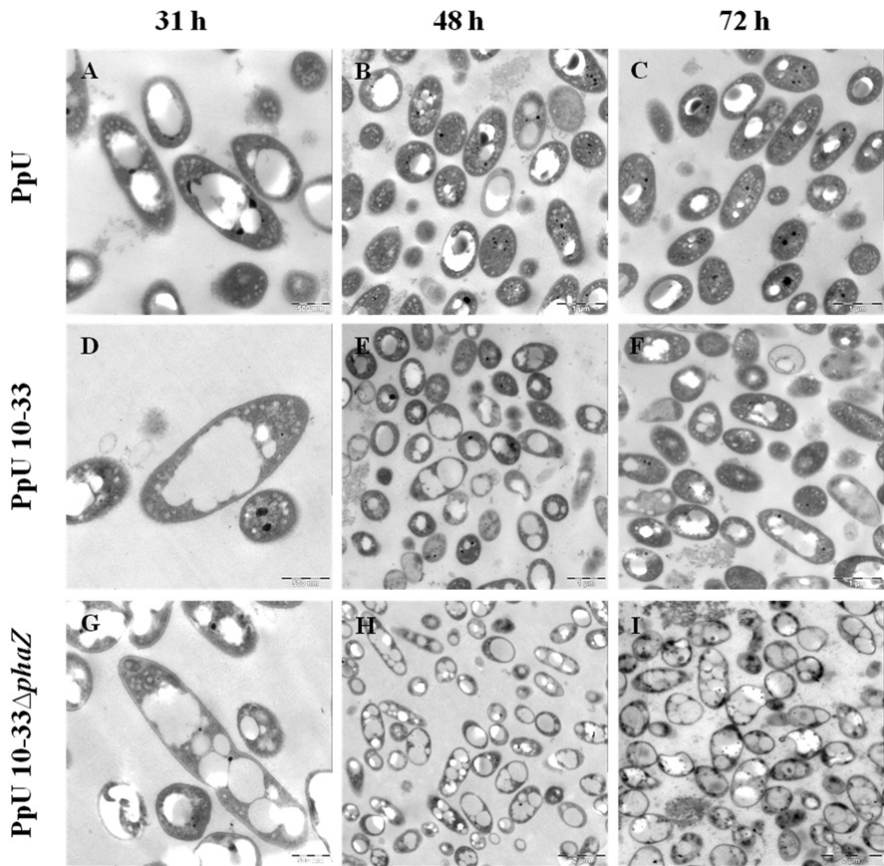
Electron micrographs of *P. putida* U wild-type and mutant cells. Transmission electron micrographs of thin sections of PpU (A–C); PpU 10–33 induced (D–F); PpU 10–33-Δ*phaZ* induced (G–I) cells. Strains were cultured in modified MM containing 35 mM sodium octanoate as a carbon source and sampled at 31 h (A, D and G), 48 h (B, E and H) and 72 h (C, F and I). Scale bars for (A, D and G), (B, C, E and F) and (H and I) are 500 nm, 1 μm and 2 μm respectively.

Since hyperexpression of PhaC2 polymerase and inactivation of PhaZ depolymerase may entrain changes in the normal cellular stoichiometry and activity of PHA proteins, and associated proteins, other changes in phenotypes may result from these genetic manipulations. To assess this possibility, we compared the ultrastructure of the PHA granules in cells of the different constructs by transmission electron microscopy (TEM). Figure 2 shows that the PpU wild-type strain (Fig. 2A–C) contains one or two defined PHA granules per cell, distributed evenly within the cytoplasm, while the PpU 10–33 *phaC2* hyperexpression strain (Fig. 2D–F) tends to contain one main granule with a morphology suggestive of the coalescence of smaller granules. This is particularly evident in the induced cultures, specifically during the mid-exponential growth phase. The *phaZ* deletion mutant tended to have multiple granules, some of which had irregular boundaries suggestive of granule fusion (Fig. 2G–I). The microscopic analysis also confirmed the results shown in Fig. 1B (Table S1B), namely that intracellular PHA accumulated in the PpU and PpU 10–33 strains starts to diminish after 48 h of cultivation, whereas the mutant lacking the depolymerase maintained accumulated PHA until the end of the experiment.

### PHAs produced by different constructs are similar but not identical

Given that the two PHA synthases of PpU have slightly different substrate specificities, with PhaC2 exhibiting a preference for 3-hydroxyhexanoyl-CoA and PhaC1 biased towards 3-hydroxyoctanoyl-CoA (Arias *et al*., 2008), it was possible that hyperexpression of the PhaC2 polymerase in PpU 10–33 might alter the monomer composition and/or physicochemical properties of the polymer produced. Table 2 shows that PHAs produced during growth on sodium octanoate by PpU, PpU 10–33 and its *phaZ* deletion mutant had similar compositions, as determined by NMR, and were copolymers of P(3-hydroxyoctanoate-*co*-3-hydroxyhexanoate), composed of 3-hydroxyoctanoate (91.4–92.5% mol) and 3-hydroxyhexanoate (7.5–8.6% mol). Also, the glass transition temperature of the three polymers, T_g_ −35.9 to −40.8°C (Table 2), was in agreement with the T_g_ described previously for medium chain length (mcl)-PHAs, and they had similar melting temperatures (T_m_, 59–61°C), indicating similar crystallinity grades.

**Table 2:** Physicochemical properties of the PHAs obtained from the mutant strains

Strains	Mw (kDa)	Mn (kDa)	PI	Tg (°C)	Tm (°C)	ΔH_m_ (J g^−1^)	Td (°C)	Monomer composition (%mol)	
								3-HHx	3-HO
PpU	126.3	76.6	1.65	−35.90	61.40	22.76	294.03	8.6	91.4
PpU 10–33 NI	132.9	75.7	1.76	−35.92	59.68	28.42	294.93	7.5	92.5
PpU 10–33 I	141.1	74.9	1.88	−37.16	59.21	24.89	294.04	8.4	91.6
PpU10-33-Δ*phaZ* NI	95.6	52.1	1.83	−40.82	59.60	27.14	293.84	8.6	91.4
PpU10-33-Δ*phaZ* I	96.2	50.1	1.92	−36.09	61.57	28.60	293.65	8.7	91.3

Experimental conditions as given in Table 1.

M_w_, weight-average molecular weight; M_n_, number-average molecular weight; PI, polydispersity index (M_w_/M_n_); T_g_ (°C), glass transition temperature; T_m_ (°C), melting temperature; T_d_ (°C), decomposition temperature; ΔH_m_ (J g^−1^), enthalpy of fusion; 3-HHx, 3-hydroxyhexanoate; 3-HO, 3-hydroxyoctanoate.

However, the polymers differed in length: the molecular weights (Mw and Mn values) of the polymers from the PpU parental strain and the PpU 10–33 (PhaC2 polymerase hyperexpressing construct) were similar, ranging from 126–142 and 74–77 kDa respectively, whereas those from the PhaZ knockout were considerably lower, 96 and 50 kDa respectively. Although this result may seem to be counter-intuitive, it is consistent with observations of Cai and colleagues (2009) and Solaiman and colleagues (2003), who also found lower polymer lengths in phaZ mutants. Thus, we cannot rule out the possibility that the depolymerase also plays a direct or indirect role in chain length determination and/or in PHA synthesis itself.

### *pha* operon transcriptional activity indicates strong coupling of polymerization and depolymerization functions over the entire growth cycle

**Figure 3:**
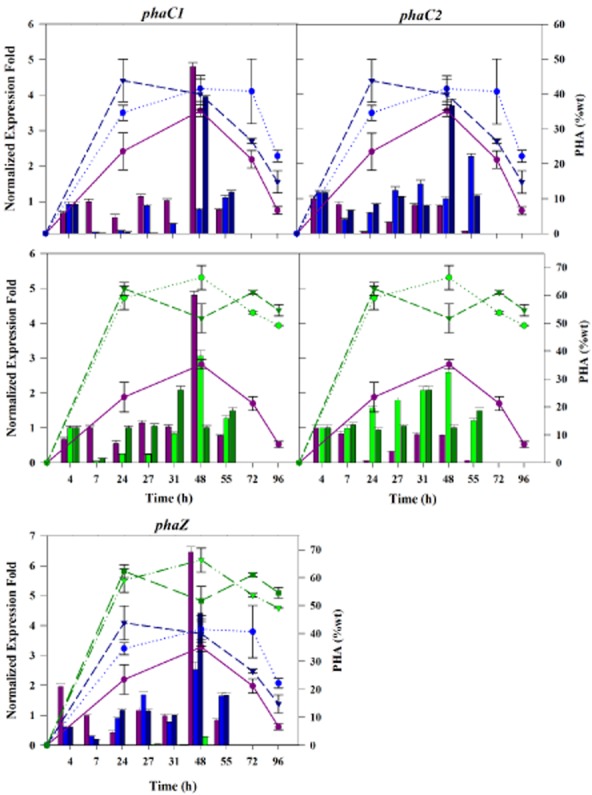
Expression of *pha* genes and PHA accumulation in *P. putida* U. Each panel shows normalized fold-increased in expression of the *pha* genes in PpU (violet), PpU 10–33 non induced (light blue) and PpU 10–33 induced (dark blue), PpU10-33-Δ*phaZ* uninduced (light green) and PpU10-33-Δ*phaZ* induced cells (dark green). The relative expression ratio of the target genes was calculated automatically with the CFX software using the standard error of the mean and the normalized expression method [ΔΔ(Ct)]. PHA content (% CDW) is showed with lines, using the same strain colour designation mentioned above. Experimental triplicates and independent biological duplicates were used for the calculations.

In order to investigate the relationship between PHA turnover and the hyperexpression of *phaC2* and *phaZ* inactivation, we also carried out transcriptional analysis by relative RT-PCR of the *pha* cluster (Figs 3 and S2), as well as of the *fadD*1 and *fadD*2 genes (Fig. S3) in the three strains. Reference genes for the RT-PCR data normalization were *gltA* and *proC2*.

### Polymerase/depolymerase gene transcripts

In the wild type, no major changes were detected in transcript levels of the two PHA polymerases, PhaC1 and PhaC2, during the first 24 h of cultivation (*P* > 0.1), and this was accompanied by a steady increase in PHA accumulation. However, a twofold increase (*P* < 0.001) in *phaZ* transcripts was measured at 4 h, corresponding to the onset of PHA production, which then fell back to lower levels. At 48 h, correlating with maximum levels of PHA accumulation, a rapid and substantive increase in the transcription of *phaC1* was observed (4.5-fold, *P* < 0.0001) and, in parallel, a sixfold increase (*P* < 0.001) in *phaZ* transcriptional activity. This was followed by a rapid decrease in the PHA content (Fig. 3), and *phaC1* and *phaZ* transcript levels. These results are indicative of a finely tuned coupling of *phaC1* transcription and PHA accumulation, on one hand, and *phaZ* transcription and PHA mobilization, on the other.

In the case of the PpU 10–33 strain, expression of the *phaC2* gene was, as expected, found to be higher than in the PpU parental strain throughout the cultivation period (*P* < 0.008) and especially at 48 h, when it peaked (3.5-fold increase, *P* < 0.0001). Interestingly, the expression of *phaC1* in this strain was mostly lower than in PpU, especially in induced cultures at 7, 24 and 48 h, suggesting that hyperexpression of *phaC2* negatively influences expression of *phaC1* (Fig. 3). However, even though hyperexpression of *phaC2* resulted in decreasing expression of *phaC1*, the combined cellular synthase activity resulted in an increased PHA production. Transcription levels of *phaZ* in PpU 10–33 tended to be similar to those in the parental strain, except at 24 h, when it was higher, correlating with the higher expression of *phaC2* and in cultures older than 48 h in which it was also higher, consistent with the higher levels of PhaC2 and PHA. There is thus also a strong coupling of PhaC2 polymerase and depolymerase synthesis.

In the PpU 10–33-Δ*phaZ* strain, significantly higher transcription levels of *phaC2* were observed throughout the cultivation period when compared with the wild type (P 0.0005-0.017), which is consistent with the higher PHA yields obtained (from 60%wt to 66%wt) (Fig. 3). In the case of *phaC1* also higher levels were measured at 24 and 38 h, but only when *phaC2* was induced (*P* < 0.0017). Thus, inactivation of *phaZ* not only prevents turnover and recycling of synthesized PHA, but also allows higher transcription levels of the PHA polymerases.

### Phasin gene transcripts

As can be seen in Fig. S2A, transcription of the *phaF* and *phaI* phasin genes in all strains increased between 4 and 7 h, when PHA started to accumulate, then temporarily decreased before spiking at 27–31 h, in the case of the PpU parental strain and the *phaZ* deletion mutant, and 48 h, in the case of the PpU 10–33 *phaC2* hyperexpression strain, when PHA accumulation was highest. Thereafter, transcription of the phasin genes decreased to low levels. Noteworthy are the much larger increases in transcription in the PpU parental strain and *phaZ* deletion mutant, compared to the PpU 10–33 hyperexpression strain (in the case of the phaZ mutant, but not the PpU 10–33 strain, this correlates with higher PHA levels), and the higher post-peak transcription levels of the PpU 10–33 mutant compared with the PpU parental strain (correlating this time with a higher PHA content). Interestingly, phasin gene expression in both the PpU wild-type strain and the *phaZ* deletion mutant spiked at 24 h well before the spiking of transcription of the *phaC1/2* polymerase and *phaZ* depolymerase genes at 48 h, whereas transcriptional activity change was more gradual in the 10–33 hyperexpression strain and tended to follow more or less PHA accumulation. The results with the wild-type strain are consistent with phasin synthesis being in phase with, but in advance of, polymer synthesis, presumably to guide correct assembly of the polymer granule.

### The PhaD *pha* gene cluster regulator

The transcription of *phaD*, which encodes a key regulator of the *pha* gene cluster, was similar in PpU and PpU 10–33 strains (except for the earliest sampling time, 4 h), but was expressed at higher levels in the *phaZ* depolymerase mutant throughout the cultivation period, consistent with the higher levels of expression of the *phaC2* polymerase and the *phaF* and *phaI* phasin genes, which PhaD regulates (Klinke *et al*., 2000; Sandoval *et al*., 2007; de Eugenio *et al*., 2010b).

### Acetyl-CoA synthase gene activities

As can be seen in Fig. S3, the transcription of the *fadD2* acetyl-CoA synthase gene exhibited a remarkable spike at 48 h, especially in the PpU wild-type strain, paralleling the sharp peak in *phaZ* transcription at the time of maximum PHA accumulation. This peak was much lower in the *phaZ* deletion mutant, which is unable to depolymerize accumulated PHA, so it would seem that the FadD2 enzyme plays a role in the recycling of monomers released by PHA depolymerization. These results corroborate those of Olivera and colleagues, which indicated that FadD2 plays a fundamental role when the PHA depolymerization process dominates in *P. putida* U (Olivera *et al*., 2001a). In contrast to the dramatic expression profile of *fadD2*, *fadD1* largely followed the expression profile of *phaD*, with higher levels at the times of initiation of PHA accumulation and maximal accumulation. Thus, FadD1 seems to be the main acetyl-CoA synthase involved in maintaining a constant supply of activated PHA precursors (see also García *et al*., 1999; Olivera *et al*., 2001a; Ruth *et al*., 2008; Hume *et al*., 2009; Ren *et al*., 2009a).

The analysis of possible transcription promoters using Softberry, PromScan and the PDBG online informatics tools, revealed that all *pha* genes are preceded by potential promoters and more specifically, that both σ^70^ and σ^54^ potential promoters were found upstream of the *phaC1* gene. A search for transcriptional terminators, using the Arnold informatics tool, also disclosed the presence of rho-independent terminators downstream of the *phaC1*, *phaZ*, *phaC2* and *phaD* genes, as well as the existence of a REP sequence (enterobacteriaceae repetitive extragenic palindromic region) downstream of *phaD* which may also act as a transcription terminator.

## Discussion

Manipulation of gene expression is widely used to investigate cellular physiology, on one hand, and to increase product yields in biotechnology, on the other (Kraak *et al*., 1997; Prieto *et al*., 1999b; Olivera *et al*., 2001b; Conte *et al*., 2006; Kim *et al*., 2006; Ren *et al*., 2009b; Arias and Bassas, 2010). Manipulation strategies may be unsuccessful *inter alia* because the real rate limiting factor has not been addressed by the strategy, often because of genetic or physiological redundancies, which can compensate for knockout/knockdown/hyperexpression strategies. When successful, they often lead to perturbation of the carefully harmonized cellular metabolic network, and may ultimately select compensatory changes in the network (Diederich *et al*., 1994). An example of quasi-redundancy is the existence of multiple PHA polymerases in many bacteria; for instance in *P. putida* U there are two PHA synthases, PhaC1 and PhaC2. Thus far, manipulations of the expression of the PHA polymerase and depolymerase genes have given inconsistent results. Several attempts to increase PHA yield through hyperexpression of the PhaC1 polymerase (Kraak *et al*., 1997; Conte *et al*., 2006; Kim *et al*., 2006; Ren *et al*., 2009b) have had only limited success, because of either instability of the plasmid constructs, or poor inducibility of the cloned gene. Other attempts, involving deletion of *phaZ* genes are more contradictory since, although in some cases the lack of PhaZ resulted in higher yields and extended accumulation of PHA during the growth cycle (García *et al*., 1999; Sandoval *et al*., 2005; Cai *et al*., 2009), in others it did not (Huisman *et al*., 1991 and Solaiman *et al*., 2003). In this work, the stable chromosomal integration of a T7 RNA polymerase-based *phaC2* hyperexpression construct led to significant increases in PHA yields. However, the elevated yields were transitory and the PHA content of cells declined dramatically after peaking at around 48 h of cultivation. This was shown to be due to the action of the PhaZ depolymerase: deletion of the *phaZ* gene in the PhaC2 hyperexpression strain resulted in higher PHA yields than the wild type and the PhaC2 hyperexpressing strains over the entire growth curve, and in maintenance of peak levels for at least 96 h. Complementation of the *phaZ* deletion resulted in lower PHA levels than the parental and hyperexpression strains, and faster PHA mobilization after 48 h. These results are in concordance with the observation of Sandoval and colleagues (2005) that PHA accumulation was not detectable in a b-oxidation mutant strain hyperexpressing PhaZ. Thus, PhaZ is clearly a rate-limiting factor in both PHA synthesis and maintenance, and would seem to be a non-redundant function in *P. putida* U. It is therefore a prime target for engineering strains for commercial production of PHA.

The implication that the PhaZ depolymerase functions not only once PHA accumulation peaks but also from the onset of PHA synthesis, was investigated by analysis of *phaZ* transcription by RT-PCR. This analysis revealed that indeed the *phaZ* gene is expressed throughout the growth curve (with an increase at the outset of PHA accumulation), as are the *phaC1* and *phaC2* genes, and its expression is correlated, in the parental strain, with the *phaC1* transcriptional level and, in the PpU 10–33 strain, with that of *phaC2*. The results presented here are thus consistent with a strong functional coupling of PHA polymerization and depolymerization activities, and with the notion that PHA polymerization and depolymerization occur simultaneously in and on the PHA granule, from the outset of granule formation, presumably constituting a key functional element of dynamic granule structuring and remodelling, and interaction with the cellular environment.

Though it was not the purpose of this study to investigate the relationships of PHA production and expression of relevant genes other than *phaC2* and *phaZ*, two observations made here would seem to merit brief comment. One is that upregulation of *phaC2* seemed to result in downregulation of *phaC1*, suggesting that, although the two PHA polymerases have non-identical substrate spectra, and are thus not entirely functionally redundant, their combined cellular levels seem to be subject to a regulatory network setting upper synthesis thresholds. Interestingly, both polymerases were expressed at higher levels in the *phaZ* knockout mutant, correlating with the higher PHA accumulation, and indicating that such a regulatory network must integrate depolymerization parameters. Secondly, the changes in levels of PhaC2 and PhaZ that we have engineered in this study, not only caused changes in PHA accumulation levels but also in PHA granule morphology. In particular, upregulation of *phaC2* resulted in larger, more amorphous granules. Since granule number and size (Pieper-Fürst *et al*., 1995; Wieczorek *et al*., 1995; Grage *et al*., 2009), as well as distribution in the cytoplasm and segregation (Ren *et al*., 2010; Galán *et al*., 2011), are orchestrated by the major PHA-associated proteins, the phasins (Steinbüchel *et al*., 1995; Prieto *et al*., 1999a; York *et al*., 1995; de Eugenio *et al*., 2010b), it would seem that upregulation of PhaC2 creates an imbalance in PHA : phasin ratios, leading to altered phasin-determined granule morphology. Such an imbalance is also indicated by the RT-PCR expression results obtained in this study: although higher expression levels of the phasins generally followed increases in PHA accumulation, but peaked prior to the peaking of expression of the PHA synthases (at 31 and 48 h respectively), peak phasin expression levels in the *phaC2*-hyperexpressing strain were lower than in the wild type. We also cannot rule out a more direct effect of any possible phasin-like activity of PhaC2 on granule morphology, though this seems unlikely in the light of the findings of Arias and colleagues (2008). It thus seems likely that this altered ratio of phasin : synthase/PHA is responsible for the fused granule morphology (see also the observations of Sandoval *et al*., 2007). In any case, these results suggest that, if a direct coupling of phasins and PHA accumulation in general, and phasins and PhaC2 synthesis in particular, exists, it may be incomplete.

In summary, the picture that emerges from this and previous studies (Ren *et al*., 2010; de Eugenio *et al*., 2010a) is that of a highly dynamic PHA granule, resulting from tight regulatory coupling of polymerization : depolymerization processes that rapidly and sensitively respond to changes in availability of carbon and other nutrients, and the physiological-metabolic needs of the cell, to ensure optimal carbon resource management. This involves continual and coordinated expression of polymerases and depolymerase, and their constant remodelling of the PHA granule, throughout the growth and stationary periods. Avoidance of futile cycles of synthesis : degradation appears to be achieved more through the control of enzyme activity, perhaps through interactions with other PHA proteins, such as the phasins, and probably through spatial partitioning of the polymerases and depolymerase, than through modulation of gene expression. In terms of the commercial production of PHA, we have shown that the PhaZ depolymerase is a key yield-limiting parameter, and that its inactivation, in combination with hyperexpression of PhaC1/2 synthases, is pivotal to obtaining high PHA yields. Our use of chromosomally integrated genetic constructs resulted in stable strains and phenotypes that obviated the need for costly antibiotic selection pressure for plasmid maintenance. Also our finding that the T7-based expression system was leaky, and did not require the addition of inducer, is a further cost-saving aspect for production.

## Experimental procedures

### Microorganisms and vectors

Bacterial strains, mutants and plasmids used in this work are summarized in Table S2.

### Culture media conditions

Unless otherwise stated, *Escherichia coli* and *P. putida* strains were cultured in Luria–Broth (LB) and incubated at 37°C and 30°C respectively. Where required, supplements were added to media as follows: rifampicin (Rf, 20 μg ml^−1^ in solid and 5 μg ml^−1^ in liquid media), kanamycin (Km, 25 μg ml^−1^ in solid and 12.5 μg ml^−1^ in liquid media), ampicillin (Ap, 100 μg ml^−1^), tellurite (Tel, 100 μg ml^−1^), gentamicin (Gm, 30 μg ml^−1^), chloramphenicol (Cm, 30 μg ml^−1^), isopropyl-β-d-thiogalactopyranosid (IPTG, 70 μM) and 5-bromo-4-chloro-3-indolyl-β-d-galactopyranoside (XGal, 34 μg ml^−1^).

### DNA manipulations

All genetic procedures were performed as described by Sambrook and Russell (2001). Genomic and plasmid DNA extraction, DNA purification from agarose gel and PCR cleaning were carried out using the corresponding Qiagen kits (QIAGEN, Hilden, Germany), according to the manufacturers' instructions. All DNA modifying enzymes were purchased from NEB (Massachusetts, USA). Polymerase chain reactions (PCR) were performed in an Eppendorf Vapo.protect Thermal Cycler (Eppendorf, Hamburg, Germany). The 50 μl PCR reaction mixture consisted of 2 μl of DNA (50 μg ml^−1^), 1× PCR buffer, 2 mM MgCl_2_ (PROMEGA, Wisconsin, USA), 0.2 μM of each primer (Eurofins mgw Operon, Ebersberg, Germany), 0.2 mM dNTPs (Amersham, GE HealthCare, Amersham, UK), and 1.25 U GoTaq Hot Start Polymerase (PROMEGA). PCR cycling conditions consist of: an initial step at 96°C/10 min, followed by 30 cycles of 96°C/30 s, 60°C/30 s and 72°C/1 min, with a final extension at 72°C/5 min. Plasmid transfer to *Pseudomonas* strains (pCNB1mini-Tn*5 xylS*/Pm::*T7*pol or pUTminiTn*5*-Tel-*phaC2*) was made by triparental conjugation experiments (Selvaraj and Iyer, 1983; Herrero *et al*., 1990) and transconjugants clones were confirmed by PCR.

PCR reactions for sequencing were performed in 10 μl reaction mixture consisted of 6–12 ng of the purified PCR product, 2 μl BigDye Ready Reaction Mix (Applied Biosystems, Foster City, CA, USA), 1 μl of BigDye buffer and 1 μl of the specific primer (2 μM). The cycling conditions included: an initial step at 96°C/1 min, followed by 25 cycles of 96°C/20 s, 52–58°C/20 s and 60°C/4 min, with a final extension step at 60°C/1 min. Nucleotide sequences were determined using the dideoxy-chain termination method (Big Dye Terminator v3.1 Kit, Applied Biosystems) and the ABI PRISM 3130 Genetic Analyser (Applied Biosystems).

Identification of potential transcriptional promoter regions and terminators was made using the Softberry, (http://linux1.softberry.com/berry.phtml), PromScan (http://molbiol-tools.ca/promscan/), BDGP online (http://www.fruitfly.org/seq_tools/promoter.html); and Arnold (http://rna.igmors.u-psud.fr/toolbox/arnold/index.php) bioinformatics tools.

### Design and construction of the *phaC2* hyperexpression strain PpU 10–33

PpU 10–33 is a *P. putida* strain U derivative in which *phaC2* gene expression is driven by the T7 polymerase promoter-T7 polymerase system (Fig. S1B). It consists of two chromosomally integrated cassettes: one containing the *phaC2* gene expressed from the T7 polymerase promoter, and another containing the T7 polymerase gene expressed from the *Pm* promoter and regulated by the cognate benzoate/toluate-inducible XylS regulator derived from the TOL plasmid. The *phaC2* cassette was constructed as follows: The *phaC2* gene of *P. putida* strain U was excised from the pBBR1MCS-3-*phaC2* plasmid (Arias *et al*., 2008), cloned into the pUC18*Not*I/T7 vector (Herrero *et al*., 1993), and the correct orientation of the gene confirmed by sequencing. The *phaC2* gene and the T7 promoter were then transferred as a cassette into the pUTminiTn*5*-Tel vector (Sánchez-Romero *et al*., 1998). First, the miniTn*5* derivative pCNB1 *xylS*/*Pm*::*T7*pol was transferred to *P. putida* U by filter-mating and selected by the Km selection marker (Harayama *et al*., 1989; Herrero *et al*., 1993). Since integration of the transposon in the genome is essentially random, and different sites of insertion can markedly influence transcription levels of inserted genes, a pool of approximately 100 transconjugants was prepared for the second transfer. A 5 ml LB culture of this pool was incubated for 3 h (30°C, 180 rpm), and used as recipient to transfer the pUTmini-Tn*5*-Tel-T7*phaC2* construct. Transconjugants were readily scored by the black colour they display when they transform the tellurite (selection marker), and subsequently confirmed by PCR. The final recipients varying in insertion sites of both cassettes were subsequently scored for PhaC2 levels and PHA, the best selected and designated PpU 10–33.

### Knockout of *phaZ* in PpU 10–33 and complementation

Deletion of the *phaZ* gene was accomplished by using a method involving a double-recombination event and selection of the required mutant by expression of the lethal *sacB* gene (Quant and Hynes, 1983; Donnenberg and Kaper, 1991). First, a DNA fragment containing the ORFs adjacent to the *phaZ* gene, encoding the PhaC1 and PhaC2 synthases, was synthesized by GENEART AG (Life Technologies, California, USA) and subsequently sub-cloned into the pJQ200SK vector containing the Gm^R^ and *SacB* selection markers. The hybrid plasmid was then introduced by triparental mating into the PpU 10–33 strain. Transconjugants, in which the plasmid was integrated into the chromosome by a single cross-over, were selected on Gm + Km + Tel-containing plates and confirmed by PCR. Deletion mutants resulting from the second recombination were subsequently selected on LB plates containing 10% sucrose, scored for sensitivity to Gm, and further analysed by PCR to confirm the position and extent of the deletion. One deletion mutant was selected and designated PpU 10–33-*ΔphaZ*.

For complementation of the deletion mutant, the *phaZ* gene (921 bp) was amplified by PCR and cloned into the pBBR1MCS-5 vector. Transconjugants were selected for their Gm resistance and further confirmed by PCR.

### RNA manipulations

Samples (3 ml) were taken from cultures through the growth phase (4, 7, 24, 27, 31, 48 and 55 h) and immediately mixed with an equal volume of RNA protect Buffer (QIAGEN). After incubation for 5 min at room temperature, suspensions were centrifuged at 5000 *g* (Allegra 25R, Beckman Coulter, California, USA), the supernatant fluids discarded, and the pellets placed at −80°C. Total RNA was extracted using the RNeasy mini kit (QIAGEN) with DNAse treatment, according to the manufacturer's protocol. RNA was then eluted in 100 μl of RNase-free water, and kept at −80°C. The integrity of the RNA was assessed by electrophoresis in formaldehyde agarose gels and the concentration and purity determined spectrophotometrically (Spectrophotometer ND-100, peQlab-biotechnologie GmbH, Erlangen, Germany). For cDNA synthesis all reagents were purchased from Invitrogen (Life Technologies) and reactions performed according to manufacturer's protocols, in 20 μl using 10 μg of total RNA and random primers. Samples in which Superscript III RT was not added were used as negative controls. After cDNA synthesis, the remaining RNA was precipitated by addition of 1/5 volume of 1 M NaOH and incubated at 65°C for 10 min, followed by 10 min at 25°C and neutralized with the same volume of 1 M KCl. The resultant cDNA was then purified using the PCR purification kit (QIAGEN) and the concentration and purity measured spectrophotometrically. cDNAs were diluted with DEPC (diethylpyrocarbonate)-treated water to 100 ng μl^−1^ and kept at 4°C.

### Relative RT-PCR assay

The MIQE guidelines for the experimental design of RT-PCR were followed (Bustin *et al*., 2009). Oligonucleotides used for the RT-PCR assays (Eurofins mgw Operon, Germany) were designed with the help of the Primer3 (http://frodo.wi.mit.edu/primer3/) and Oligo Calc (http://www.basic.northwestern.edu/biotools/oligocalc.html) bio-informatic tools and are summarized in Table S3. First, optimal PCR conditions, annealing temperature and primer concentrations were established using a standard set of samples (genomic DNA) as templates. Primer specificity was determined by melt curve analysis and gel visualization of the amplicon bands (not longer than 300 bp). Primer efficiency was determined in a dose-response assay using as a template a pool of cDNAs in a fourfold dilutions series (in triplicates) and standard curves determined for each set of primers, using the CFX96™ real-time PCR detection system (Bio-Rad, California, USA) and the CFX Manager software (Bio-Rad). In all cases, efficiencies were measured in the range between 89% and 100%. The choice of appropriate reference genes for data normalization, was determined using the geNorm method existing in the CFS software, taking into consideration the target stability for the different experimental conditions and time points. Several candidate genes, including those encoding ‘housekeeping’ (16 s rDNA, *rpsL*), general metabolism (*gltA*, *gap-1*, *proC1, proC2*), cell division (*mreB*, *ftsZ*) and signalling (*ffH*) functions, were tested and from these, *gltA* and *proC2* were selected as the reference since they showed a coefficient variance and M value of around 0.5–1. For relative RT-PCR, experimental triplicates were performed and samples without cDNA were used as negative controls. PCR reactions contained 12.5 μl of iQ™ SYBR Green Supermix (2×) (Bio-Rad), 1 μl forward primer (10 μM), 1 μl reverse primer (10 μM), 2 μl of cDNA (1/10 diluted), in 20 μl final volume. The PCR cycling conditions were: 50°C/2 min and 95°C/10 min, followed by 40 cycles of 95°C/15 s, 60°C/30 s and 72°C/30 s, with a final extension at 72°C/10 min. Fluorescence was measured at the end of each cycle. For the melting curve, an initial denaturation step at 95°C/10 min was followed by a reduction in temperature to 65°C, and then temperature increases in increments of 0.5°C/5 s up to 95°C, with continual signal acquisition.

All RT-PCR results are means of biological duplicates (from independent experiments) and experimental triplicates (of measurements), with inclusion of an internal calibrator in each plate for data normalization. For the statistical analysis, the relative expression ratio of the target genes was calculated automatically with the CFX software (Bio-Rad), using the standard error of the mean and the normalized expression method [ΔΔ(Ct)]. Values are expressed as Normalized fold-increases in expression. Two tailed unpaired student's *t*-test has been used for the statistical analysis using the GraphPad Prims5 program. *P*-values were calculated for each observation made along the test regarding differences in gene expression.

### Culture conditions for PHA production

3-methylbenzoate (3-MB) was used to induce the *Pm* promotor, via its cognate XylS activator, to mediate expression of T7 polymerase gene, which in turns triggers hyperexpression of the *phaC2* synthase. In order to determine optimal conditions for PhaC2 expression/PHA synthesis in PpU 10–33, the concentrations of the 3-MB inducer (from 0.2–3 mM), the times of induction expressed as culture density (OD_550_ 0.4–1.5), and concentrations of carbon sources in the medium, were varied and yields measured. Two-litre Erlenmeyer flasks containing 400 ml of mineral medium (MM) (Martínez-Blanco *et al*., 1990) plus 0.1% yeast extract, 15 mM sodium octanoate and appropriate antibiotics were inoculated with a colony suspension from a fresh overnight culture (incubated at 30°C) on an MM agar plate containing 20 mM succinate. Flasks were then incubated at 30°C in a rotary shaker (Multitron, INFORS AG, Switzerland) at 180 rpm. Once the cultures reached an OD_550_ of 0.8, the culture was split into two (1 l Erlenmeyer flasks containing 200 ml) and 3-MB added to a final concentration of 0.5 mM to one of both flasks. At the same time a second pulse of sodium octanoate (20 mM) was added. For the wild-type control strain, the procedure was the same but without the induction. Samples were collected every 24 h and the biomass (cellular dry weight, CDW), PHA, OD_550_, Nile red staining and NH_4_^+^ concentration were determined. For CDW determination, samples were dried at 80°C for 24 h and expressed in g l^−1^ of original culture. Values are means of duplicates of at least two independent experiments.

### Fluorescence and transmission electron microscopy

One millilitre of culture was mixed with 2 drops of a Nile red solution in dimethylsulfoxide (0.25 mg ml^−1^) and centrifuged at 16 000 *g* (Centrifuge 5417R; Eppendorf, Hamburg, Germany), at 4°C, 5 min. Pellets were washed twice with 2 ml MgCl_2_ 10 mM, and 5–10 μl of the cell suspension mounted on a microscopic slide (Bassas, 2010). The presence and morphology of PHA granules were visualized with a ZEISS Axio Imager A1 epiflourescence microscope equipped with a Cy3 filter (EX BP 550/25, BS FT 570, EM BP 605/70) (ZEISS, Jena, Germany) and the AxioVision rel 4.6.3 software (ZEISS Imaging Solutions GmbH, Jena, Germany).

For transmission electron microscopy, bacteria were fixed in growth medium containing 2% glutaraldehyde plus 5% formaldehyde at 4°C and processed as described previously (Bassas, 2010). Ultrathin sections were then examined in a TEM910 transmission electron microscope (Carl Zeiss, Oberkochen, Germany) at an acceleration voltage of 80 kV and images were taken at calibrated magnifications using a line replica and recorded digitally with a Slow-Scan CCD-Camera (ProScan, 1024 × 1024, Scheuring, Germany) with ITEM-Software (Olympus Soft Imaging Solutions, Münster, Germany).

### PHA extraction and purification

Culture samples were centrifuged at 6500 *g* for 15 min at 4°C (Allegra 25R, Beckman Coulter), and pellets washed twice in distilled water and lyophilized (Lyophilizer alpha 1–4 LSC, Christ, Germany) at −59°C at 0.140 mbar. Five millilitres of samples was taken along the growth phase to monitor the PHA production and were lyophilized. The lyophilized biomass was extracted with 10 ml chloroform for 3 h at 80°C (Bassas-Galia *et al*., 2012). PHA content (%wt) is defined as the percentage of the CDW represented by PHA.

### PHA analysis

For ^1^H and ^13^C-NMR analysis, 10–20 mg of polymer was dissolved into 0.7 ml of deuterated chloroform (CDCl_3_) and NMR spectra recorded as described elsewhere (Bassas-Galia *et al*., 2012) Chemical shifts are given in ppm relative to the signal of the solvent (^1^H: 7.26, ^13^C 77.3) and coupling constants in Hz. Standard Bruker pulse programs were used throughout. For determination of the polymers molecular weight and analysis of their thermal properties the protocol previously described by Bassas-Galia and colleagues (2012) and Cheema and colleagues (2012) was followed. Briefly, average molecular weights were determined by gel permeation chromatography in a HPLC system (Waters 2695 Alliance separations Module, Waters, USA) equipped with a Styragel HR5E column and a 2414 differential refractive index detector. Elution was with tetrahydrofuran (THF) at 45°C at a flow rate of 0.5 ml min^−1^ (isocratic). Sample concentration and injection volume were 0.5 mg ml^−1^ and 50 μl respectively. The calibration curve was obtained using a polystyrene standards kit (Fluka) in the Mw range of 10 000–700 000 g mol^−1^ (Sigma-Aldrich, Missouri, USA). The thermal properties were determined by differential scanning calorimetry (DSC), using 10–20 mg of the purified polymer for analysis. DSC analyses were performed with a DSC-30 (Mettler Toledo instruments, NY, USA). All data were acquired by STARe System acquisition and processing software (Mettler Toledo).

## Supporting information

**Fig. S1.** Genetic organization of the *pha* cluster and the expression system used for the hyperexpression of PhaC2 in PpU 10–33. **Fig. S2.** Expression of the *phaF*, *phaI* and *phaD* genes and PHA accumulation in *P. putida* U. **Fig. S3.** Expression of *fadD1* and *fadD2* genes and PHA accumulation in *P. putida* U. **Table S1.** Biomass and PHA production in *P. putida* U and the recombinant strains. **Table S2.** Strains, mutants and plasmids used in this work. **Table S3.** List of oligonucleotides used for the RT-PCR assay in this study.
