# Supplementary material for: Tight coupling of polymerization and depolymerization of polyhydroxyalkanoates ensures efficient management of carbon resources in Pseudomonas putida
Source: Microb Biotechnol. 2013 Feb 28;6(5):551–63. doi: 10.1111/1751-7915.12040 (PMC3918157; doi:10.1111/1751-7915.12040)

**Supporting information**

**Table S1. Biomass and PHA production in *P. putida* U and the recombinant strains.** (A) Biomass yields and (B) PHA accumulation (%wt). Strains were cultivated in the same conditions as mentioned in Figure 1.

**(A)**

| **Strain** | **CDW (gl^-1^)** | | | |
| --- | --- | --- | --- | --- |
|  | **24 h** | **48 h** | **72 h** | **96 h** |
| **PpU** | 1.31±0.2 | 3.07±0.15 | 2.5±0.06 | 2.13±0.07 |
| **PpU 10-33 (NI)** | 1.36±0.0 | 2.67±0.1 | 2.47±0.05 | 2.67±0.3 |
| **PpU 10-33 (I)** | 1.09±0.03 | 2.73±0.4 | 2.38±0.1 | 2.68±0.2 |
| **PpU 10-33- ∆*phaZ* (NI)** | 1.49±0.03 | 1.83±0.3 | 3.11±0.1 | 3.2±0.1 |
| **PpU 10-33 -∆*phaZ* (I)** | 1.20±0.1 | 3.05±0.3 | 3.3±0.2 | 3.25±0.2 |

**(B)**

| **Strain** | **PHA (g l^-1^)** | | | | **PHA (%wt)** | | | |
| --- | --- | --- | --- | --- | --- | --- | --- | --- |
|  | **24 h** | **48 h** | **72 h** | **96 h** | **24 h** | **48 h** | **72 h** | **96 h** |
| **PpU** | 0.32±0.1 | 1.08±0.0 | 0.53±0.05 | 0.14±0.02 | 23.5±5.3 | 35.2±1.7 | 21.2±2.5 | 6.6±1.1 |
| **PpU 10-33 (NI)** | 0.47±0.03 | 0.74±0.4 | 0.56±0.2 | 0.6±0.1 | 34.6±2.2 | 41.5±3.7 | 40.7±9.3 | 22.2±1.7 |
| **PpU 10-33 (I)** | 0.48±0.08 | 1.07±0.05 | 0.63±0.0 | 0.39±0.05 | 43.8.±6.1 | 39.85±4.4 | 26.5±0.7 | 14.8±3.18 |
| **PpU 10-33-∆*phaZ* (NI)** | 0.88±0.08 | 1.20±0.14 | 1.67±0.05 | 1.57±0.05 | 59±4.2 | 66.3±4.3 | 53.7±0.6 | 49.05±0.04 |
| **PpU 10-33-∆*phaZ* (I)** | 0.75±0.09 | 1.56±0.02 | 2.03±0.1 | 1.80±0.07 | 62.3±2.3 | 51.7±5.2 | 61±0.7 | 54.6±2.0 |

Values are means of duplicates and/or triplicates and errors were calculated with the SEM function by using the GraphPad Prism statics program.

**Table S2. Strains, mutants and plasmids used in this work.**

| **Vectors and constructions** | **Description** | **Reference** |
| --- | --- | --- |
| **RK600** | Cm^R^, *ori*ColE1, *ori*V, RK2*mob*^+^*tra*^+^. Helper plasmid in triparental conjugation events. | (Herrero *et al*., 1990) |
| **pUC18*Not*/T7** | Ap^R^, *ori*ColE1, *lacZ*α^+^, promoter *lac,* pUC18*Not*I derivative vector in which a synthetic T7 promoter sequence has been introduced from the *EcoR*I site of the polylinker. | (Herrero *et al*., 1993) |
| **pCNB1mini-Tn*5 xylS*/Pm::T7*pol*** | Km^R^, *tnp^-^*, *xylSPm* promoter, T7 RNA polymerase. | (Harayama *et al*., 1989; Herrero *et* *al*., 1993) |
| **pUTminiTn*5*-Tel** | Tel^R^, *tnp*^-^. | (Sánchez-Romero *et al*., 1998) |
| **pGEM®-T Easy** | Ap^R^, *ori*ColE1, *lac*Zα^+^, SP6 T7, promoter *lac*, Cloning PCR products vector. | PROMEGA |
| **pJQ200 (KS/SK)** | Gtm^R^, *ori*p15A, Mob^+^, *lacZ*α^+^, *sacB*, vector used for generate deletions by double recombinant events. | (Quant and Hynes, 1983) |
| **pBBR1MCS-5** | Gtm^R^, *ori*BBrl, Mob^+^, *lacZ*α^+^, promoter *lac.* Broad-host-range cloning and expression vector. | (Kovach *et al*., 1995) |
| **pBBR1MCS-3-*phaC2*** | A pGEMT Easy insert from position -26 to +1832 from the ATG of *phaC1* was cloned into pBBR1MCS-3 vector using the restriction sites *Sac*II*-Sac*I.Tc^R^. | (Arias *et al*., 2008) |
| **pUC18*Not*/T7-*phaC2*** | pUC18*Not*/T7 containing the *phaC2* excised from the pBBR1MCS-3-*phaC2* construct and cloned using the restriction site *EcoRI*. | This study |
| **pUTminiTn*5*-Tel-T7*phaC2*** | Mini-Tn5-Tel containing the T7promoter-*phaC2*- excised as a *Not*I cassette from pUC18*Not*/T7-*phaC2.* | This study |
| **pMS-*phaC1C2*-0941347** | pMS vector (spectinomycin, GENEART AG) containing a 3531 bp DNA cassette encoding the PhaC1 and PhaC2 synthases (63% G+C content). This synthetic DNA fragment was assembled from synthetic oligonucleotides and /or PCR products, and further cloned into the *Hind*III and *Kpn*I restriction sites | This study |
| **pJQ200SK-*phaC1C2*** | A synthetic DNA insert from position -106 to +3383 from the ATG of *phaC,* from the pMS-*phaC1C2*-0941347 construct**,** cloned into pJQ200SK by using the restriction site *Not*I. | This study |
| **pBBR1MCS-5-*phaZ*-1** | A pGEMT Easy insert from position -27 to + 890 from the ATG of *phaZ* cloned into pBBR1MCS-5 by using the restriction site *Kpn*I-*Xba*I. | This study |
| **Strains** |  |  |
| ***E. coli* DH5α´** | F-, Δ*lac*U169, φ80d*lac*Z1M15, *hsd*R17, *rec*A1, *end*A1, *gyr*A96, *thy*-1, λ-, *rel*A1, *sup*E44, *deo*R | Invitrogen |
| ***E. coli* DH10B** | F-*, mc*rA, Δ(*mrr*-*hsd*RMS-m*cr*BC) *Ø*80d*lac*Z1M15, Δ*lac*X74, *deo*R, *rec*A1, *end*A1, *ara*D139, Δ(*ara*, *leu*)7697, *gal*U *gal*K, λ-, *rps*L, *nup*G. | Invitrogen |
| ***E. coli* CC18λpir** | F-, Δ(*ara*-*leu*), *ara*D, Δ*lac*X74, *gal*E, *gal*K, *pho*A20, *thi*-I *rps*-1, *rpo*B, *arg*E(Amp), *rec*A, *thi pro hsd*RM+, RP4-2-Tc (CC18 lysogenied with the λpir phage) | (Herrero *et al*., 1990) |
| ***P. putida* U** | Wild type strain, Rf^R^. | (Martínez-Blanco *et al*., 1990) |
| ***P. putida* U-pCNB1mini-Tn*5xylS*/Pm::T7*pol*** | *P. putida* U containing pCNB1mini-Tn*5xylS*/Pm::T7*pol* vector. Km^R^ Rf^R^. | This study |
| **PpU 10-33** | *P. putida* U containing pCNB1mini-Tn*5xylS*/Pm::T7*pol* and pUTminiTn*5*-Tel-*phaC2*. Km^R^ Tel^R^ Rf^R^. | This study |
| **PpU 10-33∆*phaZ*** | PpU 10-33 with the *phaZ* gene knocked out. Km^R^ Tel^R^ Rf^R^. | This study |
| **pMC-*phaZ*** | PpU 10-3*∆phaZ* 3 expressing in *trans* the *phaZ* gene (pBBR1MCS-5-*phaZ*-1). Gtm^R^ Km^R^ Tel^R^ Rf^R^. | This study |

**Table S3. List of oligonucleotides used for the RT-PCR assay in this study.**

| **Gene** | **Forward Primer (5´ 3´)** | **Reverse Primer (5´ 3´)** |
| --- | --- | --- |
| **^1^16s ribosomal DNA (16s rDNA)** | ACGATCCGTAACTGGTCTGA | TTCGCACCTCAGTGTCAGTA |
| **^1^Citrate synthase (*glpA*) PP_4194** | gccgatttcatccagcatggtc | tggaccggatcttcatcctcca |
| **^1^Ribosomal protein S12 (*rpsL*) PP_0449** | GGCAACTATCAACCAGCTGGT | GCTGTGCTCTTGCAGGTTGTG |
| **^1^Glyceraldehyde 3-phosphate dehydrogenase (*gap-1*) PP_1009** | cttgaggttgacggtgaggtc | aggtgctgactgacgtttacca |
| **^1^Ffh: Signal recognition particle protein Ffh (*ffH*) PP_1461** | cggtagtcaaggatttcgtcaac | caccatcacgctctttttcttg |
| **^1^Rod shape-determining protein MreB (*mreB*) PP_0933** | cgtgaagtgttcctgatcgaag | ccgatttcctgcttgatacgtt |
| **^1^Cell division protein FtsZ (*ftsZ*) PP_1342** | cggtatctccgacatcatcaag | gagtactcacccagcgacaggt |
| **^1^Pyrroline-5-carboxylate reductase1 (*proC1*) PP_3778** | gcatttaccagccctttgaagc | caatgacgaaaggcaaatcgac |
| **^1^Pyrroline-5-carboxylate reductase 2 (*proC2*) PP_5095** | ctcccaactgaccttgcagac | gctccttatttgcccagttgttc |
| **^2^PHA synthase 1 (*phaC1*)** | GCATGTGGCCCACTTTGGC | CCCAGGTTCTTGCCCACTT |
| **^2^PHA depolymerase (*phaZ*)** | AGCAGTTTGCCCACGACTACC | GGTGGATCTTGTGCAGCCAGT |
| **^2^PHA synthase 2 (*phaC2*)** | GGCAACCCCAAGGCCTACTAC | CCGAGCGGTGGATAGGTACTG |
| **^2^Phasin PhaF (*phaF*)** | GTCAGCTTCTCGATCTGCTTGGT | GAAGAAGACGGCTGAAGATGTAGC |
| **^2^Phasin PhaI (*phaI*)** | CTCTTTGTCGATGCGTTTCTTG | CATGGCCAAAGTGATTGTGAAG |
| **^2^PhaD transcriptional regulator (*phaD*)** | GAACGTATCCACCCTGGAGATT | ATAAGGTGCAGGAACAGCCAGTAG |
| **^2^Long-chain-fatty-acid-CoA ligase 1 (*fadD1*)** | cgtgatcaagtacgtgaagaagatg | gtgaaggcgtagatgtggtacag |
| **^2^Long-chain-fatty-acid-CoA ligase 2 (*fadD2*)** | gctgtaccacatctatgccttcac | gccggagttggtgactttcag |

^1,2^Indicates that the DNA from *P. putida* KT2440 or *P. putida* U was used as a template, respectively.

**Fig. S1.** **Genetic organization of the *pha* cluster and the expression system used for the hyperexpression of PhaC2 in PpU 10-33:**(A) Shows the genetic organization of the *pha* operon in *P. putida* U; in (B) are represented the two vectors: pCNB1mini-Tn*5 xylS*/Pm::T7*pol* and pUTminiTn*5*-Tel-T7*phaC2* and it also is shown how the addition of the inductor (3-MB) triggers the expression of the *phaC2* gene driven by the T7 promoter. It is remarkable that both plasmids are integrated in the chromosome of *P. putida* U.

**(A)**


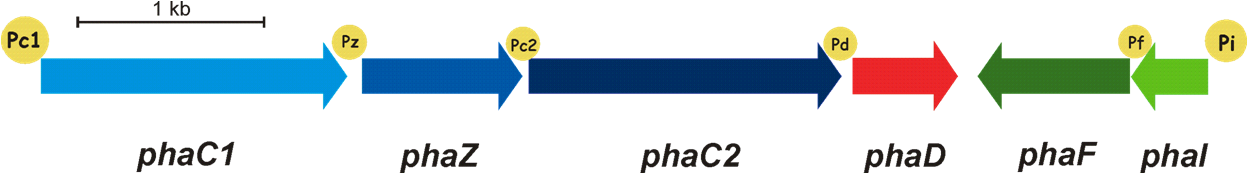


**(B)**


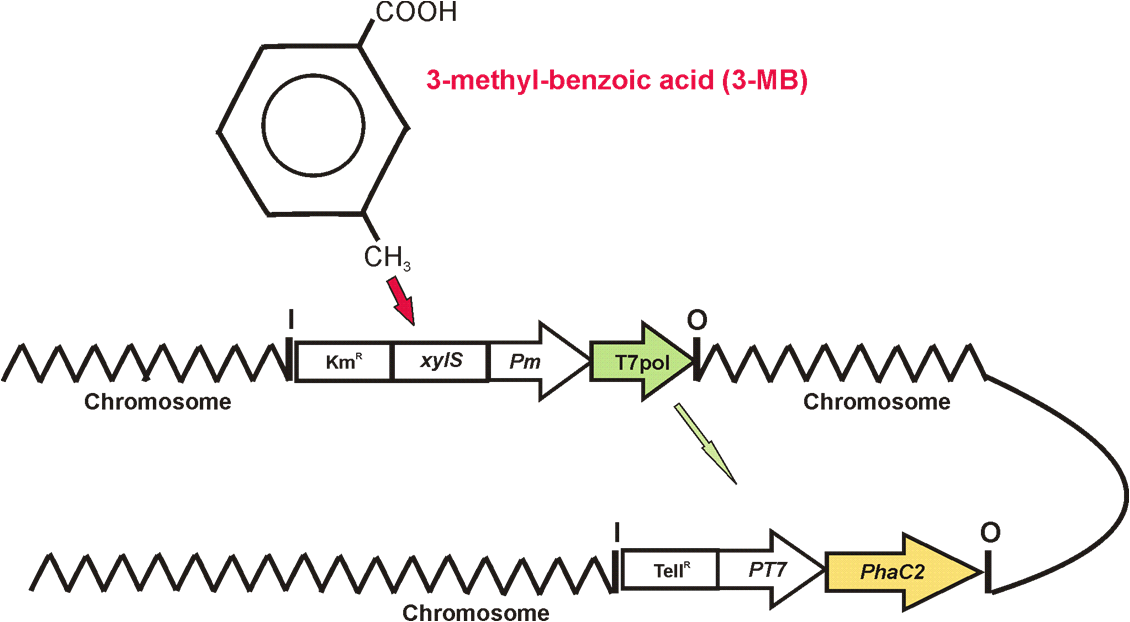


**Fig. S2**. **Expression of the *phaF*, *phaI* and *phaD* genes and PHA accumulation in *P. putida* U.** Each panel shows normalized fold-increase expression of *pha* genes when PpU (violet), PpU 10-33 non induced (light blue) and PpU 10-33 induced (dark blue), PpU10-33-Δ*phaZ* uninduced (light green) and PpU10-33-Δ*phaZ*-induced cells (dark green) were cultured in modified MM medium with octanoate (35 mM). The PHA content (% wt) is also represented with lines, using the same strain color designation mentioned above. (A) shows the normalized expression folds of *phaF* and *phaI* and (B) of *phaD*.

(A)


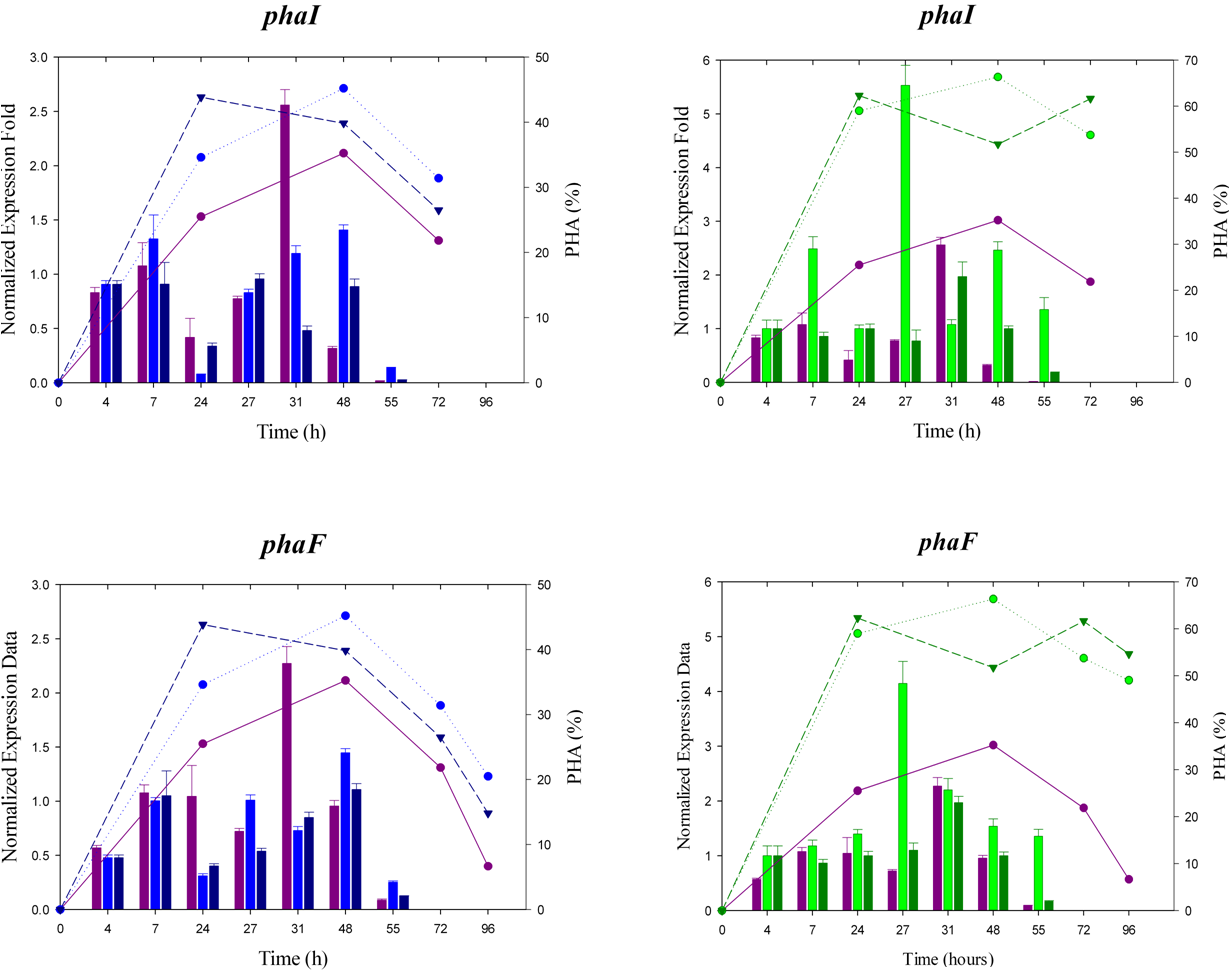


(B)


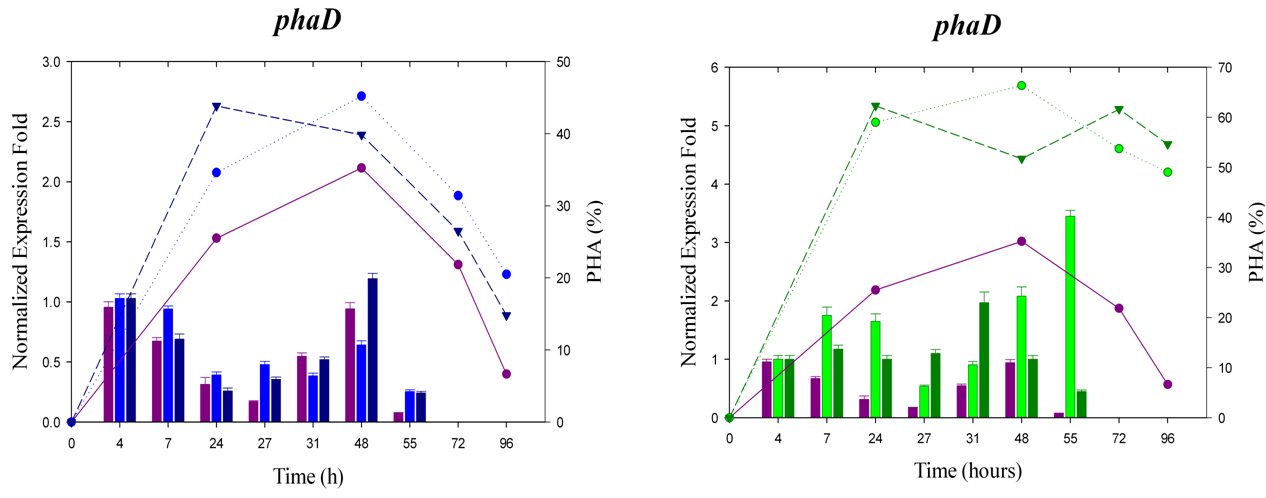


**Fig. S3**. **Expression of *fadD1* and *fadD2* genes and PHA accumulation in *P. putida* U**. Each panel shows normalized fold-increase expression of the *fadD1* and *fadD2* genes when PpU (violet); PpU 10-33 non induced (light blue) and PpU 10-33 induced cells (dark blue); PpU 10-33-∆*phaZ* uninduced (light green) and induced cells (dark green), were cultured in modified MM medium with octanoate (35 mM). The PHA content (% wt) is also represented with lines, using the same strain color designation mentioned before.


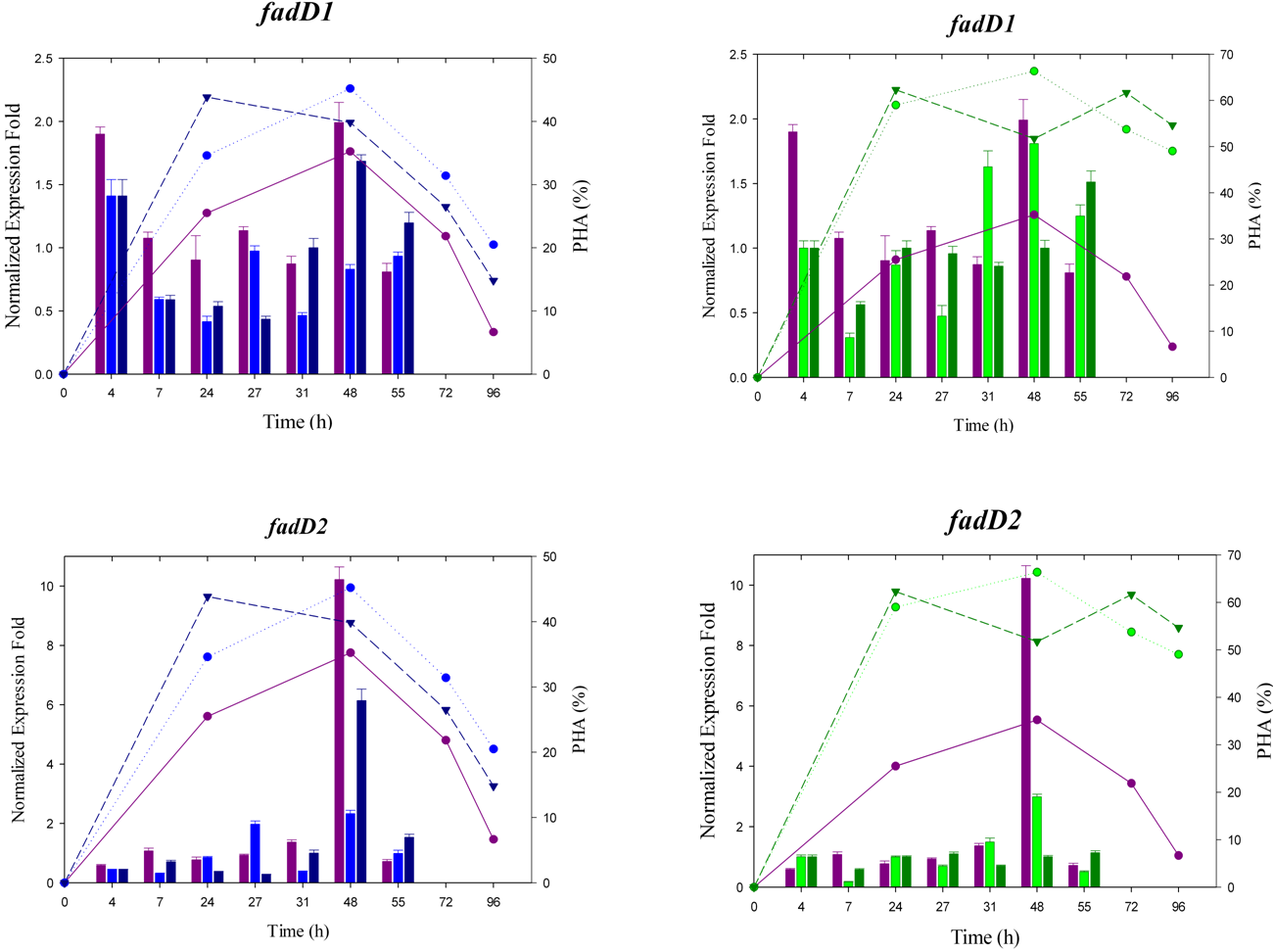

Supplement: Supplementary file 1 — Fig. S1. Genetic organization of the pha cluster and the expression system used for the hyperexpression of PhaC2 in PpU 10–33. Fig. S2. Expression of the phaF, phaI and phaD genes and PHA accumulation in P. putida U. Fig. S3. Expression of fadD1 and fadD2 genes and PHA accumulation in P. putida U. Table S1. Biomass and PHA production in P. putida U and the recombinant strains. Table S2. Strains, mutants and plasmids used in this work. Table S3. List of oligonucleotides used for the RT-PCR assay in this study. [file mbt0006-0551-sd1.doc]
